# A Novel Intraoperative CT Navigation System for Spinal Fusion Surgery in Lumbar Degenerative Disease: Accuracy and Safety of Pedicle Screw Placement

**DOI:** 10.3390/jcm13072105

**Published:** 2024-04-04

**Authors:** Haruo Kanno, Kyoichi Handa, Motoki Murotani, Hiroshi Ozawa

**Affiliations:** Department of Orthopaedic Surgery, Tohoku Medical and Pharmaceutical University, Sendai 983-8536, Japan; khanda@tohoku-mpu.ac.jp (K.H.); mmurotani@hosp.tohoku-mpu.ac.jp (M.M.);

**Keywords:** spine fusion surgery, navigation, pedicle screw, lumbar spine

## Abstract

**Background:** In recent years, intraoperative computed tomography (CT) navigation has become widely used for the insertion of pedicle screws in spinal fusion surgery. However, conventional intraoperative CT navigation may be impaired by infrared interference between the infrared camera and surgical instruments, which can lead to the misplacement of pedicle screws. Recently, a novel intraoperative CT navigation system, NextAR, has been developed. It uses a small infrared camera mounted on surgical instruments within the surgical field. NextAR navigation can minimize the problem of infrared interference and be expected to improve the accuracy of pedicle screw placement. **Methods:** This study investigated the accuracy of pedicle screw insertion under NextAR navigation in spinal fusion surgery for lumbar degenerative diseases. The accuracy of pedicle screw placement was evaluated in 15 consecutive patients using a CT grading scale. **Results:** Screw perforation occurred in only 1 of the total 70 screws (1.4%). Specifically, there was one grade 1 perforation within 2 mm, but no perforations larger than 2 mm. There were no reoperations or neurological complications due to screw misplacement. **Conclusions:** NextAR navigation can provide high accuracy for pedicle screw insertion and help ensure safe spinal fusion surgery for lumbar degenerative diseases.

## 1. Introduction

Pedicle screw fixation is a standardized and widely used surgical technique in spinal fusion surgery for lumbar degenerative diseases [1,2,3]. Conventional methods for pedicle screw insertion include the freehand technique and fluoroscopy-guided insertion. However, there is a risk of misplacement of pedicle screws which may require reoperation [4,5,6,7]. In recent years, intraoperative computed tomography (CT) navigation has become widely used for pedicle screw insertion [8,9,10,11,12]. The use of intraoperative CT navigation eliminates the need for fluoroscopic guidance, thus avoiding radiation exposure to the surgeons during screw insertion [13,14]. Pedicle screw insertion using intraoperative CT navigation is reported to be more accurate than conventional freehand or fluoroscopic insertion [4,12,15,16,17]. Additionally, intraoperative CT navigation has been shown to reduce the risk of reoperation due to misplacement of pedicle screws [9].

In conventional intraoperative CT navigation systems, an infrared camera outside the surgical field identifies the positional relationship between the patients and the surgical instruments. However, infrared interference between the camera and the surgical instruments can occasionally impair navigation guidance [11,18,19]. The infrared interference can hinder surgical technique and cause misplacement of the pedicle screw [11,18].

**Figure 1:**
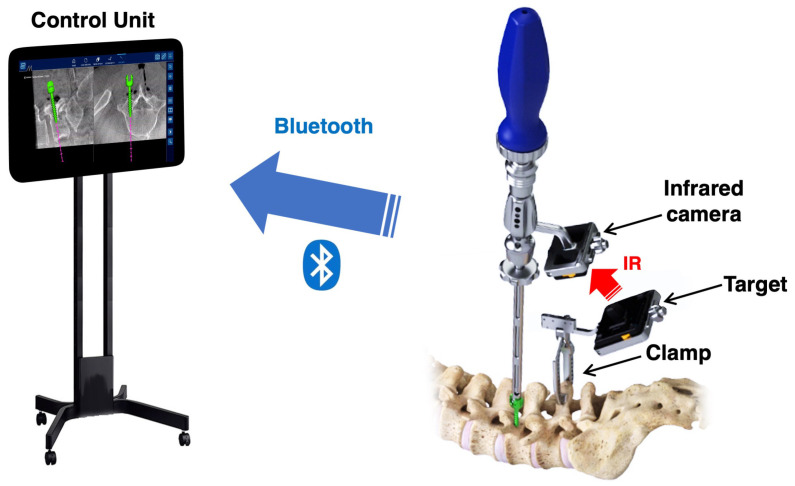
NextAR navigation system. The NextAR tracking system consists of a “target” that is firmly clamped to the spinous process and an “infrared camera” that is attached to surgical instruments (e.g., pedicle probe, tap, pedicle screwdriver). Information on the mutual positioning of the target and the camera is sent to the control unit via Bluetooth. The trajectory of the instrument on the 3D CT image is displayed in real time on the screen of the control unit. The red arrow means the infrared (IR) light from the target.

Recently, a novel intraoperative CT navigation system, NextAR (Medacta International SA, Castel San Pietro, Switzerland), has been developed [20]. It uses a small infrared camera placed on surgical instruments within the surgical field, instead of outside the surgical field (Figure 1). The NextAR navigation system minimizes the problem of infrared interference during surgery and is expected to improve the accuracy of pedicle screw insertion. However, no studies have investigated the accuracy of screw placement using the novel intraoperative CT navigation system in spinal fusion surgery. The aim of this study was to investigate the accuracy of pedicle screw insertion using the NextAR navigation system in spinal fusion surgery for lumbar degenerative diseases.

## 2. Materials and Methods

### 2.1. Patients

This study involved 15 consecutive patients (9 men and 6 women, mean age 68 years) with lumbar degenerative disease who underwent posterior instrumented fusion surgery at our institution between January and September 2023. Pedicle screw fixation was performed on all patients under intraoperative CT navigation using the NextAR system. All patients had neurological disturbance due to lumbar spinal stenosis, lumbar degenerative spondylolisthesis, or spondylolytic spondylolisthesis. The diagnosis of the lumbar degenerative disease was made based on the findings of neurological examinations and imaging studies, including plain radiograph, CT, and magnetic resonance imaging. Patients with spinal tumors, spinal infection, or congenital spinal anomaly were excluded. This study retrospectively analyzed the medical records of these patients. The Institutional Review Board of Tohoku Medical and Pharmaceutical University approved this study. This study was conducted following the principles of the Declaration of Helsinki. Written consent from the study subjects was not required as an “opt-out” process was used.

### 2.2. Navigation System

The NextAR navigation system has a unique tracking system that consists of a “target” fixed to the spinous process near the treated vertebral levels and an “infrared camera” mounted on the surgical instruments (e.g., pedicle probe, tap, pedicle screwdriver) (Figure 1). This tracking system does not require the use of infrared cameras outside of the surgical field, eliminating line-of-sight issues. Additionally, the low profile of the infrared camera and the target prevents the surgical instruments from interfering with each other when inserting pedicle screws. The control unit receives information on the mutual positioning of the target and camera via Bluetooth (Figure 1). The control unit runs the guidance software for processing the location information. The software creates a translation map between all points in the 3D CT image and the corresponding points regarding the patient’s anatomy. The trajectory of the instrument on the 3D CT image is shown in real time on the screen of the control unit. Multiple procedures such as pedicle probing, tapping, and screw insertion can be performed under navigation guidance. The data transfer via Bluetooth and data processing in the control unit is fast enough so that even quick motions of the surgeon’s hand are reflected on the navigation screen without time lag.

### 2.3. Surgical Procedure

**Figure 2:**
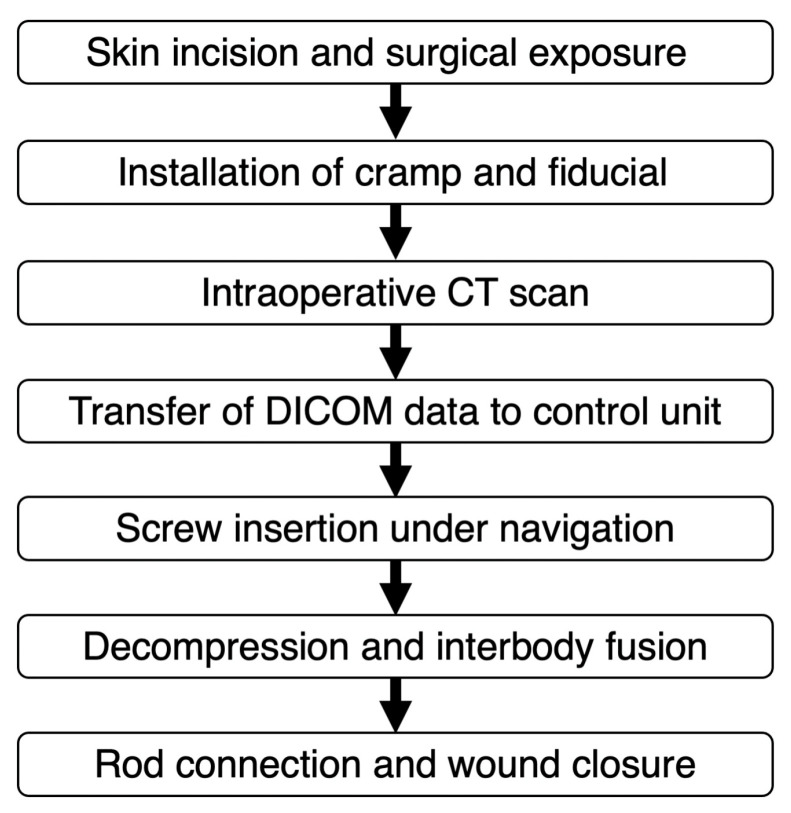
Surgical workflow.

**Figure 3:**
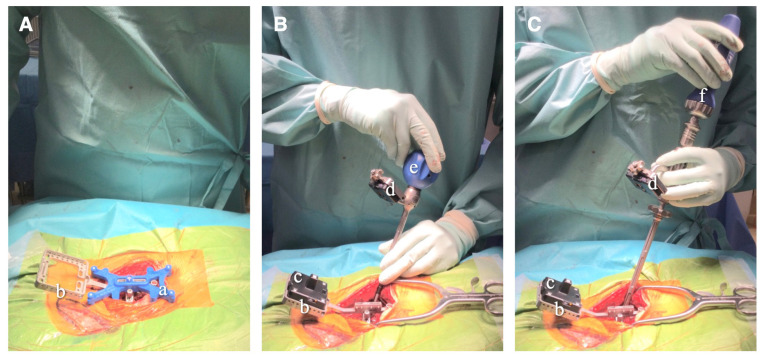
Surgical procedure for pedicle screw insertion under NextAR navigation. (**A**) During the intraoperative CT scan, the fiducial block (a) is placed over the clamp (b), which is attached to the spinous process. (**B**,**C**) The NextAR tracking system includes the target (c) mounted to the clamp (b) and the infrared camera (d) mounted on surgical instruments, such as the pedicle probe (e) and pedicle screwdriver (f).

Surgical workflow is summarized in Figure 2 (see Supplementary Materials Video S1). The patient is positioned in the prone position on a Jackson spinal table. Following a skin incision, the targeted vertebrae are exposed. A clamp is securely attached to the spinous process to mount the target. Before the intraoperative CT scan, the fiducial block is placed over the clamp (Figure 3). The guidance software defines the position of the target by computing the location of the stainless steel markers embedded in the fiducial’s propylux housing. The intraoperative CT scan is performed using the 3D C-arm (CIOS Spin, Siemens Healthineers, Erlangen, Germany). The targeted vertebrae and the fiducial must be included in the field of view (FOV) of the X-ray tube. Fluoroscopy with the 3D C-arm was used to confirm that the area to be CT scanned was appropriate in the frontal and lateral views, and then intraoperative CT was taken. The effective radiation dose during CT imaging is automatically adjusted to obtain optimal images for each patient’s body shape. After the intraoperative CT scan, the DICOM data are transferred to the NextAR control unit via a USB memory stick. Following the transfer of the DICOM data, the position of the fiducial block is automatically detected by the guidance software on the control unit. Consequently, the conventional surface registration procedure using preoperative CT [19] becomes unnecessary for navigation guidance using NextAR. In addition, there is no need to match preoperative CT data with intraoperative scan images.

**Figure 4:**
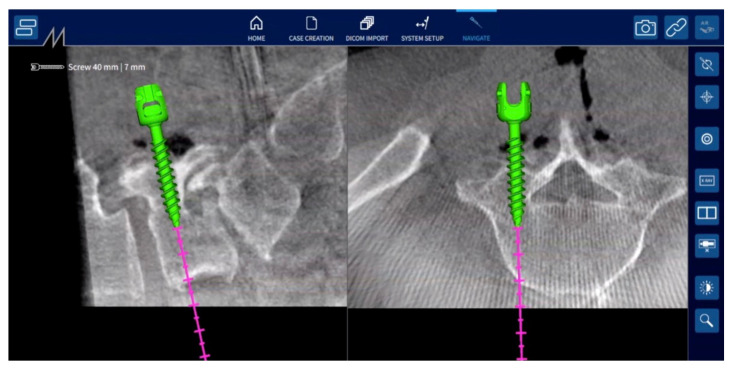
Three-dimensional CT image in NextAR navigation system. During screw insertion procedures, such as pedicle probing, tapping, and screwing, the instrument’s trajectory is displayed on the screen in transverse and sagittal planes of the 3D CT image.

Then, the fiducial can be removed from the clamp and the target can be mounted onto it. Additionally, the small infrared camera is mounted on the surgical instruments, such as the pedicle probe, screw tap, and screwdriver. Prior to beginning navigation, each instrument must be placed over the identification cavity of the clamp to be identified by the control unit. The accuracy of navigation was verified by contacting the tip of the pedicle probe with the clamp, the spinous process, and other landmarks on the bone surface. Under navigation guidance, the proper screw entry point on the bone surface is confirmed using a pedicle probe, and a screw entry hole is made using a high-speed drill. Then, a screw hole is made using a pedicle probe and screw tap, and the pedicle screw is inserted under navigation guidance (Figure 3). During the screw insertion procedures, the trajectory of the instruments is displayed on the screen in the transverse and sagittal planes of the 3D CT image (Figure 4). Hence, there is no need to use fluoroscopic guidance for pedicle screw insertion. Following the insertion of pedicle screws, surgeons perform additional procedures such as posterior decompression of neural tissue and interbody fusion to complete the surgery.

In all cases, surgery was performed using an open technique rather than minimally invasive percutaneous procedures. The screw’s diameter and length were measured and pre-planned on preoperative CT before surgery, and the pre-planned screw size was used in the surgery. Titanium alloy (Ti-6Al-4V) rods with a 5.5 mm diameter were used for pedicle screw fixation. All surgical procedures, including the pedicle screw insertion, were performed by two experienced spine surgeons in our department (H.K. and K.H.).

### 2.4. Data Collection

Baseline characteristics such as age, sex, body weight, body mass index (BMI), and clinical diagnosis were recorded. Surgical data including operative time, intraoperative blood loss, fusion levels, number of fusion levels, number of decompression levels, and operative procedure were investigated for each patient. The time required for screw insertion procedures, including probing, tapping, and screwing, was measured for each patient based on the video recording of the surgery.

The accuracy of pedicle screw placement was determined by using a CT grading scale that was previously reported [9,21]. The classification of pedicle screw perforation was divided into four grades, as follows: grade 0, no perforation of the pedicle; grade 1, perforation of 2 mm or less; grade 2, perforation from 2.1 to 4.0 mm; grade 3, perforation from 4.1 to 6.0 mm; and grade 4, perforation from 6.1 to 8.0 mm. Major perforation of the pedicle screw was defined as grade 2 or higher [9,22]. The screw perforations were evaluated using postoperative CT. The grading of pedicle screw perforation was independently assessed and blinded by an experienced spine surgeon authorized by the Japanese Orthopaedic Association. We also investigated cases of intra- or postoperative screw reinsertion and neurological complications associated with screw misplacement.

## 3. Results

Baseline characteristics for all 15 patients are summarized in Table 1. The diagnoses of the patients were lumbar spinal canal stenosis in 7 cases, lumbar degenerative spondylolisthesis in 6 cases, and lumbar spondylolytic spondylolisthesis in 2 cases. Two of these fifteen cases were complicated by thoracic myelopathy associated with a degenerative spine. One case had restenosis of the spinal canal after lumbar decompression surgery.

Surgical data for all cases are summarized in Table 2. All 15 patients underwent pedicle screw fixation and posterolateral fusion after decompression in the lumbar spine. Transforaminal lumbar interbody fusion (TLIF) using a titanium cage was added in 11 of 15 cases. Laminectomy for thoracic myelopathy was also added in two patients. The operative time was 257 ± 62 min, and the intraoperative blood loss was 296 ± 121 mL. The fusion levels were L3–L4 in one case, L3–L5 in two cases, L4–L5 in three cases, L4–S1 in three cases, and L5–S1 in six cases. The number of fusion levels was 1.3 ± 0.5. The number of decompressed levels was 2.7 ± 1.4. A total of 70 pedicle screws were inserted in the patients in this study. The number of inserted pedicle screws was 4 at L3, 18 at L4, 28 at L5, and 20 at S1. The screw insertion time, including probing, tapping, and screwing, was 25 ± 10 minutes for each patient. Screw insertion time per screw was 5.3 ± 1.5 min.

In the assessment of screw perforation, 69 screws (98.6%) showed no perforation (grade 0), while only 1 screw (1.4%) had a perforation within 2 mm (grade 1). There were no major perforations assigned to grade 2 or higher. Grade 1 screw perforation occurred medially at the L5 vertebral level in the 10th case out of 15 during the study period. There were no cases of intraoperative or postoperative screw reinsertion. The perioperative complications associated with screw misplacement were not observed. There was no difference in screw insertion accuracy between the two surgeons. In this series, no mismatch between camera and target of the NextAR system occurred that would have reduced the accuracy of screw insertion in any cases.

## 4. Illustrative Cases

**Figure 5:**
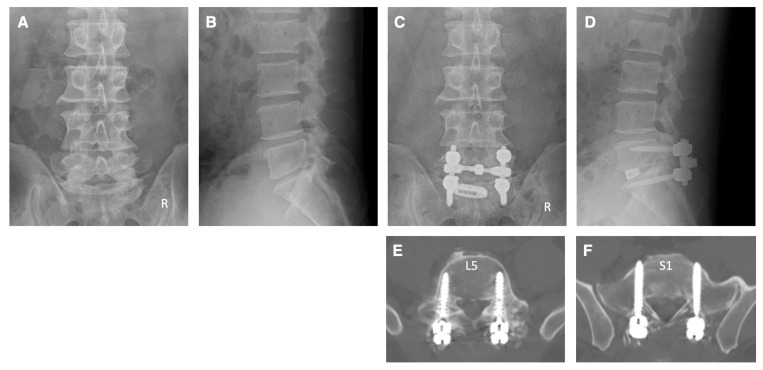
Illustrative case 1. (**A**,**B**) Preoperative X-ray shows narrowing of the disc space at the L5–S1 levels. (**C**,**D**) Postoperative X-ray shows pedicle screw fixation and TLIF at L5–S1 levels. (**E**,**F**) Postoperative CT images show that the pedicle screws were inserted appropriately.

Case 1: A 42-year-old man came to our hospital with numbness in his left lower extremity and difficulty walking. The patient had L5 and S1 radiculopathy due to lateral recess stenosis and foraminal stenosis on the left side at the L5–S1 levels. Pedicle screw fixation was performed at L5–S1 levels under navigation guidance using NextAR. Decompressions of the spinal canal, left foramen, and TLIF were also performed at the L5–S1 levels. Postoperative X-ray and CT clearly showed that the pedicle screws were inserted appropriately (Figure 5). The tip of the pedicle screw at the S1 level was properly and safely penetrating the cortical bone of the promontorium of the sacrum on the CT image (Figure 5). After surgery, the numbness of the left lower limb had improved to normal, and he was able to walk without any support.

**Figure 6:**
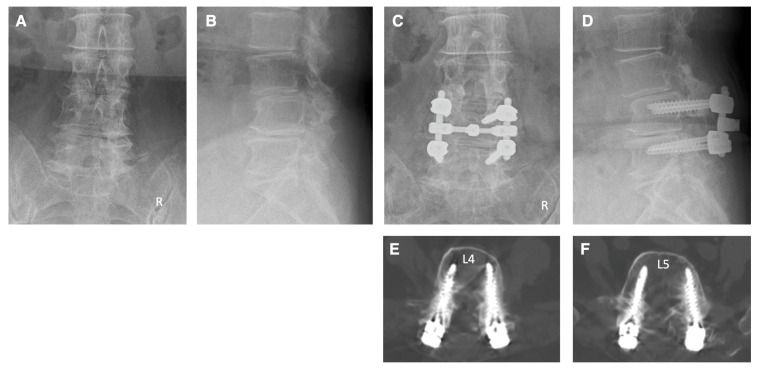
Illustrative case 2. (**A**,**B**) Preoperative X-ray shows degenerative spondylolisthesis at the L3 and degenerative changes at multiple levels. (**C**,**D**) Postoperative X-ray shows pedicle screw fixation at the L4–5 levels and posterior decompression at L2–5 levels. (**E**,**F**) Postoperative CT images show that the pedicle screws were appropriately inserted.

Case 2: A 74-year-old woman came to our hospital with numbness in both of his lower extremities and intermittent claudication. She was diagnosed as having cauda equina syndrome and right L4 radiculopathy due to spinal canal stenosis at the L2–5 levels, foraminal stenosis on the right side at the L4–5 levels, and degenerative spondylolisthesis at the L3. The patient underwent pedicle screw fixation at the L4–5 levels under navigation. Additionally, decompressions of the spinal canal at L2–5 levels and foramen on the right side at the L4–5 levels were performed. Postoperative X-ray and CT images show that the pedicle screws were appropriately inserted (Figure 6). Following the surgery, there was a significant improvement in the numbness of the lower limbs and intermittent claudication.

**Figure 7:**
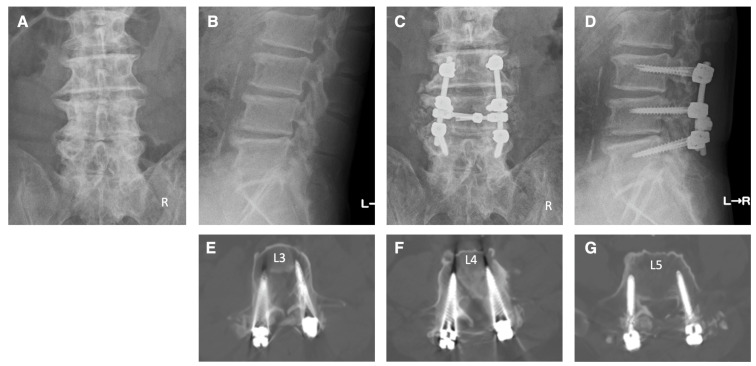
Illustrative case 3. (**A**,**B**) Preoperative X-ray shows facet joint hypertrophy and spur formation due to degenerative changes at multiple levels in the lumbar spine. (**C**,**D**) Postoperative X-ray shows pedicle screw fixation at the L3–5 levels and posterior decompression at L2–5 levels. (**E**–**G**) Postoperative CT images show that the pedicle screws were appropriately inserted.

Case 3: A 68-year-old male patient came to our hospital with pain and numbness in his left anterior thigh, as well as intermittent claudication. The patient had a medical history of posterior herniotomy for disc herniation at the L4–5 and L5–S1 levels over 10 years ago. We diagnosed the patient with left L3 and L4 radiculopathies due to spinal canal stenosis at the L2–5 levels and foraminal stenosis on the left side at the L3–5 levels. The patient underwent pedicle screw fixation at the L3–5 levels under navigation. Additionally, posterior decompressions were performed on the spinal canal at levels L2–5 and on the left side foramen at levels L3–5. Postoperative X-ray and CT images indicate that the pedicle screws were properly inserted (Figure 7). Subsequent to the surgery, there was a marked improvement in the pain, numbness, and walking difficulties of the patient.

**Figure 8:**
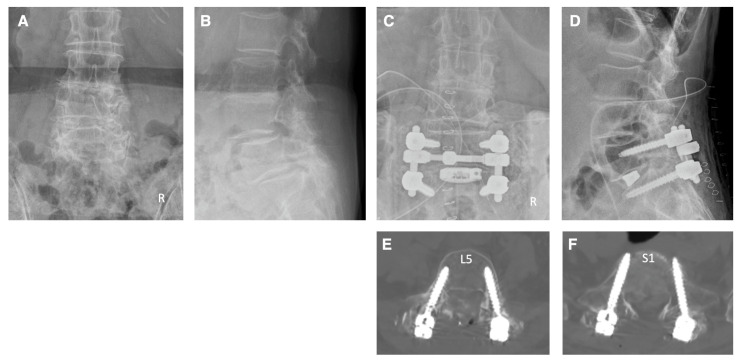
Illustrative case 4. (**A**,**B**) Preoperative X-ray shows degenerative spondylolisthesis at the L4 and L5. (**C**,**D**) Postoperative X-ray shows pedicle screw fixation and TLIF at L5–S1 levels and posterior decompression at the L4–S1 levels. (**E**,**F**) Postoperative CT images show that the pedicle screws were inserted appropriately.

Case 4: The patient, a 75-year-old woman, complained of pain and numbness in her right lower extremity, as well as intermittent claudication. Her neurological diagnosis was L5 radiculopathy on the right side. Imaging studies revealed spinal stenosis at L4–S1 and foraminal stenosis on the right side at the L5–S1 levels. The patient underwent pedicle screw fixation at the L5–S1 levels under navigation. Spinal canal decompression at L4–S1 levels and right foraminal decompression and TLIF at L5–S1 levels were also performed. Postoperative X-ray and CT scans confirmed appropriate insertion of the pedicle screws (Figure 8). The screw tip at the S1 level penetrated appropriately the cortical bone of the sacral promontorium on the CT image (Figure 8). Following surgery, the patient’s symptoms in the right lower limb improved and she was able to walk normally.

## 5. Discussion

Previous studies have shown that the perforation rates for pedicle screws range from 6% to 31% with freehand insertion and 8% to 19% with fluoroscopic guidance [4]. In contrast, the perforation rate of conventional intraoperative CT navigation is reported to be between 0% and 11% [4]. Intraoperative CT navigation is considered the most accurate method for screw insertion. This study found that the screw perforation rate under intraoperative CT navigation using the NextAR system was only 1.4%. This finding suggests that NextAR navigation may provide higher accuracy of pedicle screw insertion compared to conventional CT navigation. It has been reported that the clinically problematic perforations of pedicle screws are those that are greater than 2 mm [9,23]. In the results of this study, there were no perforations larger than 2 mm. Furthermore, there were no reoperations or neurological complications due to screw misplacement. Therefore, the novel CT navigation system using NextAR is expected to enhance the safety of spinal fusion surgery.

Previous studies have indicated that the freehand technique for inserting pedicle screw requires a long learning curve [24,25]. In contrast, a previous study has shown that the learning curve for inserting pedicle screws under intraoperative CT navigation is short [26]. This study evaluated the accuracy of screw insertion during the initial period of our NextAR navigation-guided surgery for lumbar degenerative diseases. In the results of this study, there were no major screw perforations graded 2 or higher. Furthermore, only one grade 1 screw perforation occurred in the 10th case out of the 15 cases. Notably, no screw perforation occurred at the beginning of our use of the NextAR navigation system in spine surgery. These results indicate that the learning curve for pedicle screw insertion using NextAR navigation may be short in spinal surgery for lumbar degenerative diseases.

Spine surgery navigation systems typically use infrared light, which can cause line-of-sight issues that interfere with the surgical procedure [11]. To ensure proper navigation guidance, a linear trajectory is required between the surgical instruments and reference arc in the operative field and the infrared camera outside the operative field. Improper camera or reference arc positioning can cause the line-of-sight interruption, making proper navigation impossible [11,18,19]. To avoid these issues, the infrared camera and reference arc must be placed in appropriate positions. In addition, the surgeon must be constantly aware of the position of the surgical instruments and the location of the infrared camera during surgical procedures. Consequently, the surgeon’s attention may shift from the surgical field [11,19]. In contrast, the NextAR navigation system uses the infrared camera mounted on the surgical instruments inside the surgical field, minimizing line-of-sight issues. In addition, there is no infrared camera outside the surgical field, allowing the surgeon to concentrate on the procedure in the surgical field. These systemic advantages can enhance the precision of screw insertion and simplify surgical techniques under navigation guidance.

Recently, various instrument tracking systems have been reported, including navigation [27,28], robotics [28,29,30,31], and augmented reality [27,31,32], for pedicle screw insertion in spine surgeries. These instrument tracking systems could improve the accuracy of pedicle screw placement [29,30]. However, these systems normally have an infrared camera or other type of camera outside the surgical field to identify the surgical instruments [27,28]. Thus, as with conventional navigation systems, these instrument tracking systems also can create the line-of-sight issues to interrupt the surgical procedures. If the novel instrument tracking system of NextAR is applied to the next generation of robotics and augmented reality technology, it could improve the accuracy and safety of pedicle screw insertion and other surgical procedures in spine surgeries.

The increased use of fluoroscopic imaging during surgery poses a risk to the surgeons due to ionizing radiation exposure. The harmful effects of radiation exposure on surgeons include direct exposure to the hands and indirect exposure to radiosensitive organs such as the lens of the eye, thyroid gland, and reproductive organs due to scattered radiation. In recent years, there have been many reports on the issues of intraoperative radiation exposure to spine surgeons [33,34,35,36,37]. Importantly, the use of intraoperative CT navigation eliminates the need for fluoroscopic guidance, thus avoiding radiation exposure to the spine surgeons during pedicle screw insertion [13,14]. In fact, in all patients included in this study, intraoperative CT navigation with NextAR allowed for safe screw insertion without any fluoroscopic guidance.

The conventional preoperative CT-based navigation system requires the preparation of patient-specific CT data before surgery [19]. In addition, preoperative CT-based navigation also requires a surface registration procedure during surgery to match the preoperative CT image with the patient’s anatomy. If the surface registration procedure is performed inaccurately, navigation accuracy is compromised [19]. In contrast, the intraoperative CT navigation can eliminate the complex procedures associated with preoperative CT-based navigation. In this study, the data of intraoperative CT image were transferred to the NextAR control unit, and navigation guidance was accurate during screw insertion. Therefore, the use of an intraoperative CT navigation system offers various clinical benefits, such as decreased radiation exposure and simplified surgical procedures in spinal fusion surgery.

## 6. Conclusions

This study evaluated the accuracy of pedicle screw insertion using the NextAR intraoperative CT navigation system. Out of the 70 screws inserted, only 1 (1.4%) resulted in screw perforation, which was a grade 1 perforation within 2 mm. No perforations larger than 2 mm were observed, and there were no reoperations or neurological complications due to screw misplacement. These findings suggest that NextAR navigation can achieve high accuracy in pedicle screw insertion, which may contribute to the safety of spinal fusion surgery for lumbar degenerative diseases.

## Figures and Tables

**Table 1:** Baseline characteristic in all participants.

Baseline Characteristics	
Age (years)	68 ± 11
Sex	
Male (*n*)	9
Female (*n*)	6
Height (cm)	160 ± 12
Body weight (kg)	64 ± 14
BMI (kg/m^2^)	25 ± 5
Diagnosis	
Lumbar spinal canal stenosis (*n*)	7
Lumbar degenerative spondylolisthesis (*n*)	6
Lumbar spondylolytic spondylolisthesis (*n*)	2

**Table 2:** Surgical data in all participants.

Surgical Data	
Operative time (min)	257 ± 62
Intraoperative blood loss (mL)	296 ± 121
Fusion levels (*n*)	
L3–L4	1
L3–L5	2
L4–L5	3
L4–S1	3
L5–S1	6
Number of fusion levels	1.3 ± 0.5
Number of decompression levels	2.7 ± 1.4

## Data Availability

The datasets used and/or analyzed during this study are available from the corresponding author upon reasonable request.

## Supplementary Materials

The following supporting information can be downloaded at: https://www.mdpi.com/article/10.3390/jcm13072105/s1, Video S1: Surgical procedures using a novel intraoperative CT navigation system for spinal fusion surgery in lumbar degenerative disease.
